# Mapping brain tumor microstructure: A multimodal study of diffusion MRI, intraoperative fluorescence, and neuropathology in navigated biopsies

**DOI:** 10.1016/j.nicl.2025.103921

**Published:** 2025-12-12

**Authors:** Elisabeth Klint, Johan Richter, Teresa Nordin, Ida Blystad, Martin Hallbeck, Alexandra Golby, Carl-Fredrik Westin, Karin Wårdell

**Affiliations:** a Department of Biomedical Engineering, Linköping University, Linköping, Sweden; b Department of Neurosurgery, Linköping University Hospital, Sweden; c Department of Radiology in Linköping, and Department of Health, Medicine and Caring Sciences, Linköping University, Linköping, Sweden; d Centre for Medical Image Science and Visualization (CMIV), Linköping University, Linköping, Sweden; e Department of Clinical Pathology in Linköping and Department of Biomedical and Clinical Sciences, Linköping University, Linköping, Sweden; f Department of Neurosurgery, Brigham and Women’s Hospital, Harvard Medical School, Boston, MA, USA; g Department of Radiology, Brigham and Women’s Hospital, Harvard Medical School, Boston, MA, USA

**Keywords:** 5-aminolevulinic acid (5-ALA), Diffusion MRI, Fluorescence spectroscopy, Frameless navigated brain tumor biopsy, Neuropathology

## Abstract

High-grade glioma characteristics such as heterogeneity and diffuse growth present a major diagnostic and therapeutic challenge, making accurate imaging essential for diagnosis and surgical planning. Diffusion MRI (dMRI) shows promise in tissue identification through a negative correlation between the dMRI apparent diffusion coefficient and tumor cellularity. Further, tissue disorganization due to tumor growth is correlated with decreased fractional anisotropy (FA) from diffusion tensor imaging (DTI). Q-space trajectory imaging (QTI) through free gradient waveform encoding during dMRI acquisition has been suggested as a framework for dMRI scalar map generation, enabling disentangled measures of shape, size, and orientation. We aimed to extend a clinically integrated workflow for optical guidance in frameless navigated brain tumor biopsies to include DTI and QTI scalars for multimodal analysis. Diffusion scalars were compared to tumor indications on tissue fluorescence, conventional imaging, and neuropathology in navigated brain tumor biopsy procedures.

In seven high-grade glioma patients, the biopsied tissue volume was associated with decreased dMRI features (anisotropy, kurtosis, and order parameters) and increased diffusivity in DTI when compared with contralateral white matter. Principal components of diffusion scalars depend on microstructural (QTI) and diffusivity (DTI) parameters, respectively. Redundancy analysis between the dMRI scalars revealed scalar pairs that offer novel information for tissue separation that could be of interest for fine-tuning of the MRI protocol before further evaluation of QTI for tumor tissue identification in the clinical setting.

## Highlights

• Q-space trajectory imaging can be useful in high-grade glioma tumor identification.
• Biopsy volumes characterized by decreased anisotropy, kurtosis and order parameters.
• Lack of trend between scalars and tissue fluorescence indicate different mechanisms.
• Redundancy analysis found microscopic, macroscopic, and diffusivity scalar groups.

## 1 Introduction

High-grade gliomas present a major diagnostic and therapeutic challenge due to their heterogeneity, diffuse growth, and infiltrative nature. These structural complexities make accurate imaging essential for diagnosis, surgical planning, and targeted therapy. However, tumorous tissue often extends beyond what is visible on conventional imaging (Lunsford et al., 1986, Watanabe et al., 1992, Price and Gillard, 2011).

To better delineate tumor margins preoperatively, diffusion MRI (dMRI) has emerged as a promising tool for assessing tissue microstructure (Alexander et al., 2019, Nilsson et al., 2018). The temporal scale over which water diffusion is captured in dMRI corresponds to the time scale of molecular displacement. In turn, the spatial displacement during this time matches the spatial scale of microstructural features in brain tissue (Cercignani et al., 2018). This alignment makes dMRI particularly well-suited for noninvasively probing tissue architecture. Thus, many dMRI sequences have been proposed for brain tumor assessment over the last decade. The apparent diffusion coefficient (ADC) (Le Bihan et al., 1986) is routinely acquired in clinical settings and has been shown to correlate with tumor cellularity (Nilsson et al., 2018, Chen et al., 2013, Ellingson et al., 2010). Similarly, diffusion tensor imaging (DTI) (Basser et al., 1994) metrics such as fractional anisotropy (FA) have been associated with tissue disorganization resulting from tumor growth, axonal loss, and neuronal death (Padhani et al., 2009). However, FA reflects both diffusion anisotropy and orientation coherence, making it a composite measure that lacks specificity (Szczepankiewicz et al., 2015). To better characterize diffusion behavior, Westin et al. introduced Q-space trajectory imaging (QTI), a mathematical framework based on the fourth-order covariance tensor (Westin et al., 2016). QTI enables the disentanglement of size, shape, and orientation contributions into macroscopic and microscopic anisotropy (C${}_{M}$, FA, C${}_{\mu }$, μFA), orientation coherence (C${}_{c}$), and size variance (C${}_{MD}$), among other parameters.

While QTI offers enhanced biophysical specificity, its clinical adoption has been limited by demanding postprocessing requirements and a lack of validation against established intraoperative or neuropathological methods.

In parallel, intraoperative optical techniques such as microscopy, spectroscopy, and wide-field imaging have been used to identify tumor tissue but typically provide feedback only after tissue extraction (Valdés et al., 2016). To address this limitation, our group developed an optical probe system for frameless navigated biopsies that enables in situ measurements of protoporphyrin IX (PpIX) fluorescence. PpIX accumulates selectively in high-grade glioma tissue (positive predictive value: 89%–100%, negative predictive value: 22%–91% (Hadjipanayis et al., 2015)) after oral administration of 5-aminolevulinic acid (5-ALA) due to the disruption of the blood–brain barrier and metabolic changes in tumor cells, such as a more acidic environment and altered enzyme concentrations (af Klinteberg et al., 1999, Collaud et al., 2004, McCracken et al., 2022). The in situ PpIX fluorescence measurements were linked to preoperative MRI through neuronavigation (Klint et al., 2023). Integration and postoperative correction of the biopsy trajectory allow voxel-wise comparison between MRI-derived tissue characteristics and detailed neuropathological analysis of the sampled region. This methodology, combining fluorescence with conventional MRI, has been evaluated in a study focusing on fluorescence (Klint et al., 2024) and quantitative MRI relaxometry (Klint et al., 2025). In the present study, we extended the analysis to include diffusion MRI. Specifically, we investigated the microstructural characteristics of high-grade glioma tissue in navigated brain tumor biopsy patients using diffusion MRI (DTI and QTI), intraoperative PpIX fluorescence, and neuropathology, and evaluated diffusion metrics with potential clinical value for guiding tumor sampling and optimizing MRI protocols.

## 2 Methods

### 2.1 Patients

Nine patients (mean age 67 years, range 39–79 years, two women) referred for navigated brain tumor needle biopsy at Linköping University Hospital were included in the study. Suspected tumor locations were frontal, temporal, or multilobular, involving the insula, basal nuclei, or corpus callosum. Ethical approval was granted (EPN 2020-01404), and written informed consent was obtained from each patient. In preparation for fluorescence guidance, an oral dose of 5-aminolevulinic acid (5-ALA) (20 mg/kg, Gliolan®, Medac, Germany) was administered 2-3 h before surgery (Hadjipanayis and Stummer, 2019).

Conventional imaging and fluorescence data from five patients were previously published, where the focus was on optical (Klint et al., 2024) and MR relaxometry data (Klint et al., 2025), respectively.

**Fig. 1:**
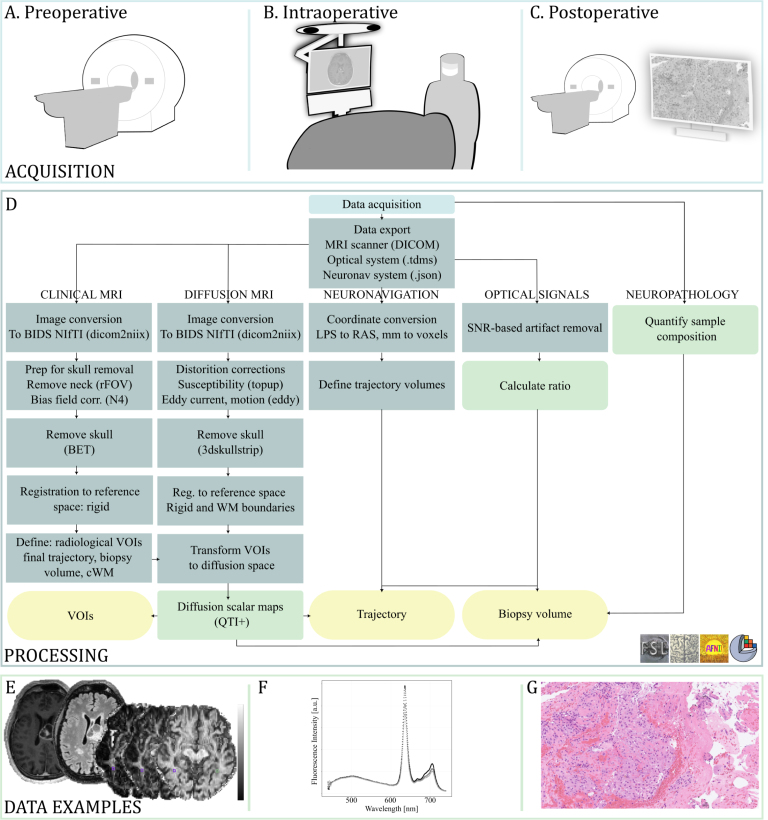
Data acquisition and processing with data examples. (A) Preoperative MRI acquisition of conventional sequences as well as diffusion MRI through diffusion tensor imaging (DTI) and q-space trajectory imaging (QTI). (B) Intraoperative acquisition of optical data and neuronavigation coordinates. (C) Postoperative MRI and neuropathological analysis. (D) Processing pipeline from data acquisition to data quantification for analysis. A combination of open-source software libraries, including FMRIBs Software Library (FSL), Advanced Normalization Tools (ANTs), Analysis of Functional NeuroImages (AFNI), and 3D Slicer, was used. (E) Example MR images (from left) contrast-enhanced T${}_{1}$w, T${}_{2}$w fluid attenuated inversion recovery, diffusivity, and anisotropy scalar maps. (F) Fluorescence spectrum with PpIX-peak at 635 nm. (G) Neuropathology slide stained with Hematoxylin and Eosin. BIDS: Brain Imaging Data Structure; cWM: contralateral white matter; FOV: field of view; SNR: signal-to-noise ratio; VOI: volume of interest.

### 2.2 Image acquisition

Pre-, intra-, and postoperative data acquisition, processing workflow, and data examples are illustrated in Fig. 1. Routine imaging for tumor assessment was acquired on a 3T MR scanner (Prisma or Skyra, Siemens Healthineers, Erlangen, Germany) equipped with a 20-channel head coil. The protocol includes T${}_{1}$-weighted (w) imaging, T${}_{2}$w, T${}_{2}$w-fluid attenuated inversion recovery (FLAIR), as well as repeated T${}_{1}$w imaging after administration of gadolinium (Gd) contrast enhancement (Gd dose: 0.2 ml/kg, Dotarem, 279 mg/ml). In addition to the clinical protocol, two diffusion sequences were acquired before administration of contrast enhancement. One high-angular resolution diffusion imaging (HARDI, DTI) 2D echo-planar imaging (EPI) sequence based on the Stejskal and Tanner pulse sequence (b-values ≤ 2000 s/mm${}^{2}$, voxel size 2.0 ×2.0× 2.0 mm${}^{3}$). Another 2D EPI free gradient waveform q-space trajectory imaging (QTI) (Westin et al., 2016) sequence was added. The latter incorporates linear, planar, and spherical diffusion encodings (b-values ≤ 2000 s/mm${}^{2}$, voxel size 2.2 ×2.2× 4.0 mm${}^{3}$). For both diffusion sequences, b${}_{0}$ images with opposite phase encoding were acquired for distortion correction. Acquisition details are listed in Table 1.

Postoperatively, a standard T${}_{1}$w image was acquired to confirm the final trajectory and biopsy sampling volume.

**Table 1:** Image acquisition parameters.

Sequence	Voxel size (recon)	FOV	TE	TR	TI	Acc	b-values	Directions	Acquisition
	[mm${}^{3}$]	[mm${}^{2}$]	[ms]	[ms]	[ms]	(ref lines)	[s/mm${}^{2}$]	[#]	[min:s]
T${}_{1}$w 3D GRE	1.0 ×1.0× 1.0	256 × 176	2.26	2300	900	GRAPPA	–	–	5:20
						2 (24)			
T${}_{1}$wGd 3D GRE	1.0 ×1.0× 1.0	256 × 176	2.26	2300	900	GRAPPA	–	–	5:20
						2 (24)			
T${}_{2}$w 3D SE	0.9 ×0.9× 1.0	256 × 176	407	3200	–	GRAPPA	–	–	4:50
						2 (24)			
T${}_{2}$w FLAIR 3D SE	1.0 ×1.0× 1.0	256 × 176	388	5000	1600	GRAPPA	–	–	4:50
						3 (24)			
HARDI 2D EPI	2.0 ×2.0× 2.0	220 × 220	94	4300	–	2 (32)	0–2000	103	7:50
(dMRI)								+ PA	+ 0:20
QTI 2D EPI	2.2 ×2.2× 4.0	244 × 244	115	4500	–	2 (30)	L: 0–2000	34	2:50
(dMRI)							P: 0–2000	19	1:40
							S: 0–1400	19	1:40
								+PA	+ 0:20

Acc: Acceleration technique; dMRI: diffusion MRI; EPI: echo-planar imaging; FLAIR: fluid attenuated inversion recovery; FOV: field of view; GRAPPA: GeneRalized Autocalibrating Partial Parallel Acquisition; GRE: gradient echo; HARDI: high-angular resolution diffusion imaging; L: linear; P: planar; S: spherical; PA: posterior–anterior; QTI: q-space trajectory imaging; SE: spin echo; TE: echo time; TI: inversion time; TR: repetition time.

### 2.3 Surgical procedure with fluorescence-guidance

The clinical navigated biopsy procedure was pursued with the addition of optical measurements through the biopsy needle as it is inserted toward the planned biopsy position. The biopsy needle was modified with an aperture at the tip of the outer cannula to allow forward-looking measurements. The intended sampling position is hereafter referred to as ‘target’ (0 mm).

The procedure is described in detail in Klint et al. (2023). In brief, the biopsy target and trajectory were planned on preoperative structural images in the neuronavigation system (StealthSystem S8, Medtronic Inc., USA). The images were registered to the patient’s anatomy through tracing of the head and scalp using a surface matching algorithm. The Autoguide® (Medtronic Inc.) was configured as support during needle insertion, and the optical probe was functionally tested against a sterile test plate. The skull and dura mater were opened. Insertion of the probe-in-needle kit was made in 1–14 mm steps. In each position, real-time measurements of tissue characteristics were recorded and displayed. Near the planned target, the location with the highest PpIX-peak was located, and the sampling window of the biopsy needle was adjusted to overlap the identified area. The outer cannula of the probe was held in place, the probe was taken out, and the inner cannula was inserted. Biopsy specimens were sampled in four directions and sent to a neuropathologist for intraoperative smear analysis according to clinical routine.

### 2.4 Data processing

#### 2.4.1 Neuropathology and fluorescence

Diagnosis was set according to the 2021 WHO CNS Tumor Classification Guidelines (W.H.O. Classification of Tumours Editorial Board, 2021) within 14 days of surgery. Tissue specimens were fixed in formalin, embedded in paraffin, and sectioned into 3μm slabs. The sections were stained with Hematoxylin and Eosin (H&E) (Fig. 1G) and Ki67 before digitization. Molecular analysis of IDH1/2 mutation (Therascreen IDH1/2 RGQ PCR kit, QIAGEN) and MGMT-methylation status (Therascreen MGMT Pyro kit, QIAGEN) were routinely performed. If clinically motivated for diagnosis, massive parallel sequencing (Genomic Medicine Sweden 560 panel for solid tumors, GMS560) was performed. Within the framework of the project, all slides were reviewed by a senior neuropathologist (M.H.), and coarse percentages of high- and low-grade tumor, necrosis, infiltrative zone, and non-tumor tissue presence in the specimens were reported.

Tissue fluorescence was reported as a binary measure of PpIX-peak (+) or no peak (-) (Fig. 1F). A peak was defined as a ratio > 0.1 between the intensity at maximum emission wavelength (λ: 635 nm) divided by intensity at maximum tissue autofluorescence (λ ≈ 500 nm) (Haj-Hosseini et al., 2010, af Klinteberg et al., 1999). The intensity at the maximum emission wavelength was corrected for the contribution from other tissue fluorophores through exponential fitting.

#### 2.4.2 Neuroradiology

Structural images were corrected for bias field inhomogeneities, skull stripped, and rigidly registered (Advanced Normalization Tools Avants et al., 2011; FMRIB’s Software Library (FSL) Jenkinson et al., 2012) to the T${}_{1}$w contrast-enhanced image. Diffusion images were corrected for distortions from Eddy currents, magnetic susceptibility, and EPI using the diffusion preprocessing commands in FSL. The transformation matrix between conventional and diffusion space was calculated through linear registration together with white matter boundary matching (‘epireg’ in FSL). A detailed overview of the processing scheme is found in Fig. 1D.

White matter (WM), cortical gray matter (GM), and cerebrospinal fluid (CSF) were delineated in SynthSeg+ (Billot et al., 2023). A senior neuroradiologist (I.B.) segmented contrast-enhancing tumor, necrosis, and edema volumes in the structural images. Segmentation was based on initial region-growing (‘Segment Editor’, 3D Slicer) (Fedorov et al., 2012). All volumes of interest (VOIs) were transformed into the two dMRI spaces. Tissue label conflicts were resolved through subtraction of the contrast-enhancing tumor, necrosis, and edema from WM, GM, and CSF volumes. The final trajectory was defined by needle impact hypointensities in the postoperative image. The biopsy volume and a 10 mm in diameter reference in the contralateral WM were segmented with regard to the final trajectory. The placement of the contralateral WM volume was determined by a senior neuroradiologist based on the position of the biopsy volume and the presence of normal-appearing WM. If the immediate contralateral tissue did not fulfill these two criteria, the closest normal-appearing WM of sufficient diameter was chosen instead.

Diffusion scalar maps were derived from the QTI+ framework using semidefinite programming to impose positivity constraints on the diffusion and covariance tensors (Herberthson et al., 2021, Boito et al., 2022). Calculated maps included measures of diffusivity (MD, AD, RD, C${}_{MD}$), anisotropy (macroscopic: C${}_{M}$, FA; microscopic: C${}_{\mu }$, μFA), kurtosis (MK, MKt, K${}_{bulk}$, K${}_{\mu }$, K${}_{shear}$), orientation (C${}_{c}$, OP, OP2), and variance (V${}_{iso}$, V${}_{MD}$, V${}_{shear}$) presented in Westin et al. (2016) (see examples in Fig. 1E).

#### 2.4.3 Multimodal comparisons and statistics

Assessments were made on three levels: biopsy volume compared to contralateral WM, measurement points along the needle trajectory, and radiological VOIs (yellow boxes in Fig. 1B).

Between the biopsy volume and the contralateral WM, a glioma ‘feature vector’ was constructed from clinical images, diffusion scalar distributions, PpIX-peak occurrence, tumor percentage, and Ki67 index. Diffusion scalar mean values and their standard deviation were assessed using two-sided t-tests assuming equal variance. Corrections for type 1 error through False Discovery Rate (FDR) with the Benjamini–Hochberg method were made (p<0.05). Relative distribution changes were represented with arrows (↑, ↓) or lines (-) signifying increase, decrease, or no change, respectively. Principal component analysis (PCA) with logistic regression was implemented to elucidate the relative contribution of each scalar.

Along the needle trajectory, per-patient mean and standard deviation (m ± s.d.) diffusion scalar values were assessed in relation to PpIX-peak occurrence and tissue type on conventional imaging.

In the radiological VOIs, one-way ANOVA and post-hoc Tukey’s HSD were used to compute separation between tissue types (CSF, GM, WM, contrast-enhancing tumor, edema, and necrosis) based on the diffusion scalar maps. Pairwise correlations of mean VOI values were assessed through Pearson’s correlation metric, as suggested by Villani and colleagues (Villani et al., 2022). Across-tissue redundancy matrices were calculated. A scalar pair with an absolute correlation greater than 0.5 across all tissues and patients was considered redundant.

**Fig. 2:**
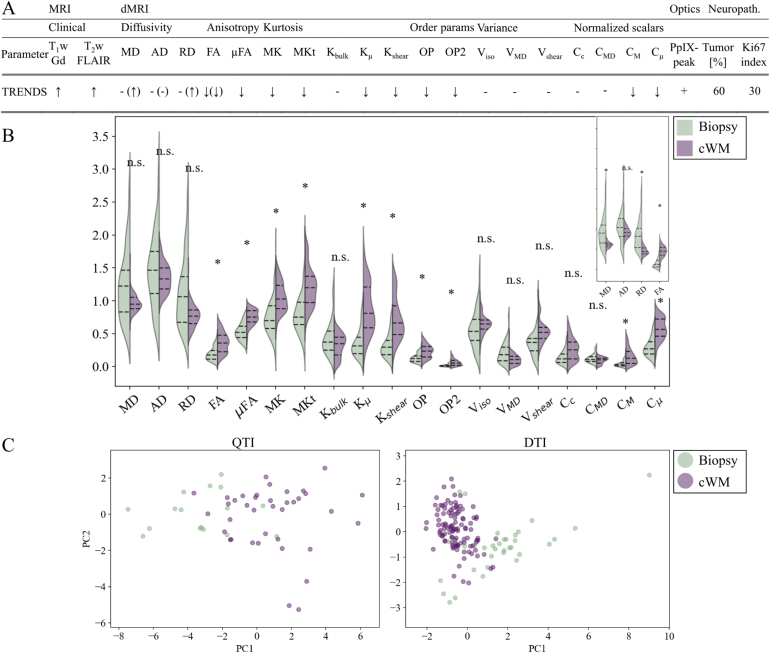
High-grade glioma feature vectors in the biopsy volume compared to the contralateral normal-appearing white matter. (A) Parameter trends on conventional images (contrast-enhanced T_1_w, T_2_w fluid attenuated inversion recovery (FLAIR)), diffusion scalar maps (diffusivity, anisotropy, kurtosis, orientation, variance, and normalized scalars), protoporphyrin IX (PpIX) peak presence, and tumor percentage, as well as proliferation index (Ki67) from neuropathology. ↑ denotes increase, ↓ decrease, and - no difference. Symbols in parentheses denote diffusion tensor imaging (DTI) trends. (B) Violin plot of diffusion scalar map distributions in the biopsy volume (left) and contralateral white matter (right). The main plot depicts q-space trajectory imaging (QTI) data, and the inset in the upper right corner illustrates DTI data. Asterisks (*) denote level of significance. (C) Principle components and test set data for QTI (left) and DTI (right). Scalar maps: diffusivity (mean: MD, axial: AD, radial: RD), anisotropy (macroscopic: FA; microscopic: μFA), kurtosis (mean: MK, MKt, K${}_{bulk}$, microscopic: K${}_{\mu }$, K${}_{shear}$), order parameters (OP, OP2), variance (isotropic: V${}_{iso}$, V${}_{MD}$, V${}_{shear}$) and normalized scalars (C${}_{c}$, C${}_{MD}$, C${}_{M}$, C${}_{\mu }$). Gd: gadolinium; neuropath: neuropathology; n.s.: non-significant; params: parameters; PC: principal component.

## 3 Results

Neuropathological diagnoses were seven high-grade gliomas, one lymphoma, and one non-tumor. The subsequent analysis focused on the high-grade gliomas.

### 3.1 Biopsy volume feature vector

High-grade glioma tissue was represented by: contrast enhancement on T${}_{1}$wGd, hyperintensity on T${}_{2}$w FLAIR; decreased anisotropy, kurtosis, and orientation parameters; PpIX fluorescence peaks; majority tumorous tissue, and Ki67 index of 30% (Fig. 2A). The biopsy volume showed significantly lower anisotropy (C${}_{M}$, FA, C${}_{\mu }$, μFA), kurtosis (MK, MKt, K${}_{\mu }$, K${}_{shear}$), and orientation alignment (OP, OP2) than contralateral WM, as well as a significant increase in DTI MD and RD (Fig. 2B). Fluorescence peaks were present in all high-grade glioma biopsy volumes. Tumor percentages were 20%–95%, with the majority of high-grade glioma tissue in all but two cases: one dominated by infiltrative tumor tissue and one by necrosis. For details from the neuropathological analysis, see Table S1 in the Supplementary material. Similar trends of decreasing scalar values were seen in the lymphoma and non-tumor cases.

QTI and DTI distribution trends were consistent, however, resulted in a significant increase in MD and RD in DTI while the difference did not reach significance in QTI. Additionally, absolute diffusivity values were consistently higher in QTI compared to DTI (Fig. 2B and Table S2 in the Supplementary material). The largest contribution in the first principal component was scalars probing microscopic features (C${}_{\mu }$, K${}_{\mu }$, and μFA) in QTI and diffusivity (MD, and RD) in DTI. The second component was influenced by kurtosis and variance (C${}_{MD}$, K${}_{bulk}$, and V${}_{shear}$) for QTI and anisotropy (FA) for DTI (Fig. 2C).

**Fig. 3:**
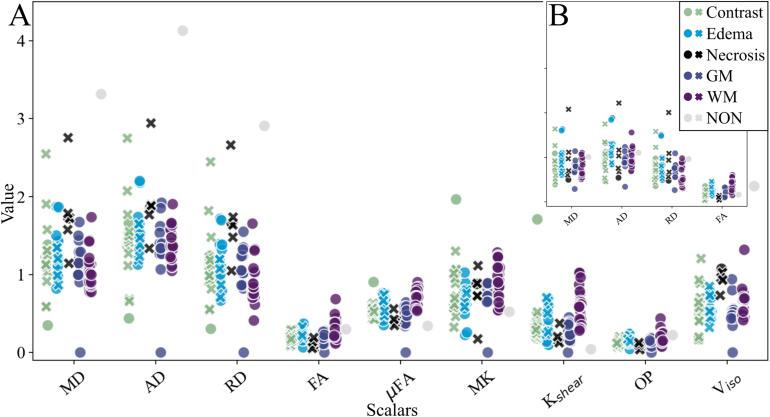
Mean scalar values and protoporphyrin IX fluorescence in each measurement position along the trajectory. (A) Sample of q-space trajectory imaging (QTI) scalars (see Fig. S1 in the Supplementary material for additional scalars). (B) Diffusion tensor imaging (DTI) scalars. Scalar maps: diffusivity (mean: MD, axial: AD, radial: RD), anisotropy (macroscopic: FA; microscopic: μFA), kurtosis (MK, K${}_{shear}$), orientation parameters (OP), variance (V${}_{iso}$). Crosses: PpIX-peaks, circles: no peak. GM: gray matter, WM: white matter.

### 3.2 Needle trajectory

Intraoperatively, 86 measurement positions were assessed along the needle trajectory in patients with high-grade gliomas. PpIX-peaks were present in tissue defined as contrast-enhancing tumor, edema, and necrosis on conventional imaging. Fig. 3 presents mean scalar values in each measurement point per tissue type in relation to PpIX-peak presence. No pattern was discovered between scalar values and fluorescence.

### 3.3 Radiological volumes of interest

**Fig. 4:**
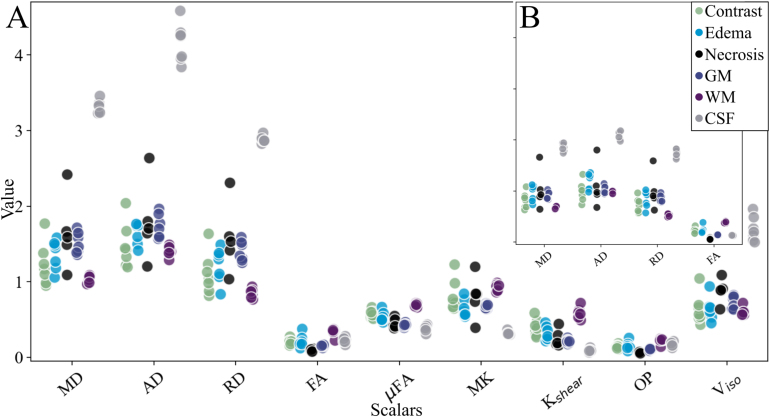
Mean scalar values per patient in each radiologically defined tissue type (contrast-enhanced tumor, edema, necrosis, GM, WM, and CSF). (A) Sample of q-space trajectory imaging (QTI) scalars (see Fig. S2 in the Supplementary material for additional scalars). (B) Diffusion tensor imaging (DTI) scalars. (C) Normalized parameters (C${}_{c}$, C${}_{M}$, C${}_{MD}$, C${}_{\mu }$) per tissue type represented on the unit circle. Scalar maps: diffusivity (mean: MD, axial: AD, radial: RD), anisotropy (macroscopic: FA; microscopic: μFA), kurtosis (MK, K${}_{shear}$), order parameters (OP), isotropic variance (V${}_{iso}$). CSF: cerebrospinal fluid, GM: gray matter, WM: white matter.

Mean scalar values in CSF, GM, and WM, together with tumor-associated tissue volumes (contrast-enhancing tumor, edema, and necrosis), are presented in Fig. 4. Scalar values for contrast-enhancing tumor and edema were inseparable in all scalars, exemplified by the mean of the normalized diffusion scalars (C${}_{c}$, C${}_{M}$, C${}_{MD}$, C${}_{\mu }$) visualized on the unit circle in Fig. 4C. Note that none of the scalar values exceed 0.5, i.e., reside in the lower half of the available range (0–1). Similar trends were observed for QTI and DTI data. Analogous to the biopsy volume analysis, lower parameter values were observed with the DTI method than for QTI (Fig. 4B).

**Fig. 5:**
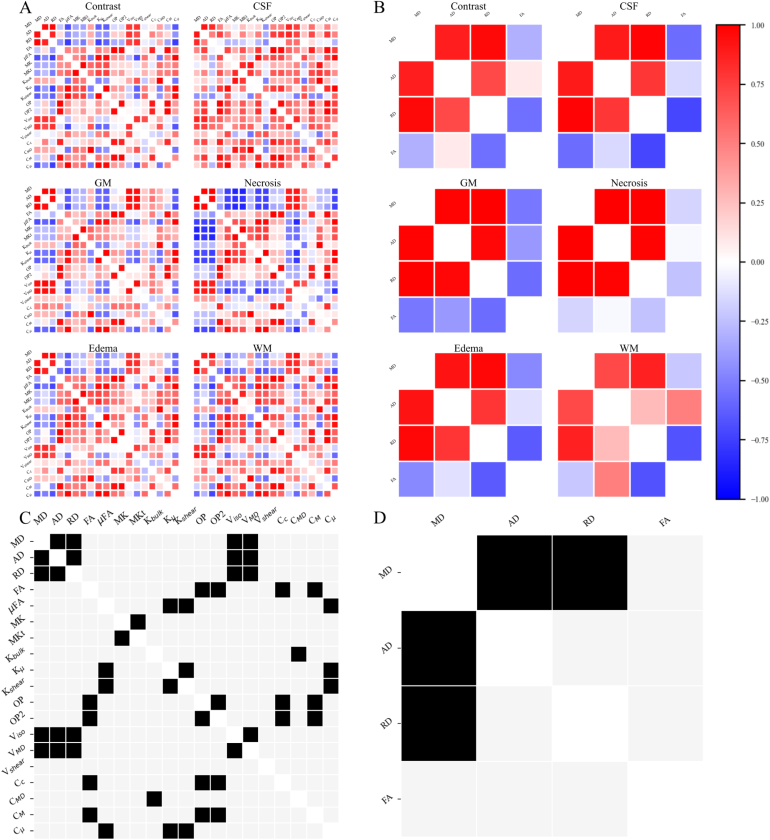
Diffusion scalar correlation and redundancy matrices. Pearson’s correlation of (A) Q-space trajectory imaging (QTI) and (B) Diffusion tensor imaging (DTI) scalars. Redundancy matrix for all tissue types for (C) QTI, and (D) DTI data. Note that the 4 × 4 matrices from DTI are reflected in the upper left quadrant of each QTI matrix. Scalar maps: diffusivity (mean: MD, axial: AD, radial: RD), anisotropy (macroscopic: FA; microscopic: μFA), variance (isotropic: V${}_{iso}$, V${}_{MD}$, V${}_{shear}$), kurtosis (mean: MK, MKt, K${}_{bulk}$, microscopic: K${}_{\mu }$, K${}_{shear}$), order parameters (OP, OP2), and normalized scalars (C${}_{c}$, C${}_{MD}$, C${}_{M}$, C${}_{\mu }$). CSF: cerebrospinal fluid, GM: gray matter, WM: white matter.

Pearson’s correlations per scalar pair and tissue type are depicted in Fig. 5. In general, positive correlation between diffusivity (MD, AD, RD) and variance (V${}_{iso}$, V${}_{MD}$); macroscopic anisotropy (C${}_{M}$, FA) and order parameters (OP, OP2); and between microscopic anisotropy (C${}_{\mu }$, μFA) and kurtosis (K${}_{\mu }$, K${}_{shear}$) were identified. Negative correlation between diffusivity and anisotropy was observed throughout the QTI and DTI data, except for CSF in the QTI data, where almost exclusively positive correlations were observed. These cross-tissue type correlations were further emphasized in the redundancy matrices, see Fig. 5C and 5D.

## 4 Discussion

### 4.1 High-grade glioma features

We present a dMRI feature vector for high-grade glioma biopsy volumes indicating decreasing trends in anisotropy, kurtosis, and order parameters in high-grade glioma tissue compared with contralateral WM. Decreased anisotropy (FA) has previously been suggested to correlate with tumor cellularity (Padhani et al., 2009, Kinoshita et al., 2008) and tumor infiltration (Padhani et al., 2009). In their 2015 study, Szczepankiewicz and colleagues showed the added benefit of disentangling cell shape from FA through μFA (DIVIDE: Diffusional Variance Decomposition) (Szczepankiewicz et al., 2015). They demonstrated low FA and μFA in glioma, while μFA was high in meningioma. In the present study, decreased FA and μFA were found in all biopsy volumes (high-grade glioma, lymphoma, and non-tumor) compared to contralateral WM. Further, the first principal component for QTI saw its greatest contribution from microscopic features.

### 4.2 Tumor indications outside contrast enhancement

In addition to μFA differences in contrast-enhancing tumor, decreased μFA was also found in edema and necrosis. As brain tumor cells are known to extend beyond contrast enhancement on clinical MRI (Lunsford et al., 1986, Watanabe et al., 1992, Price and Gillard, 2011), especially along white matter tracts, the possibility of tumor infiltration cannot be excluded. However, other potential influences include partial volume effects due to the large slice thickness of the diffusion images, in particular QTI. On the other hand, the large slice thickness limits the sensitivity of detecting small infiltrative zones.

PpIX-peaks have also been reported outside contrast enhancement (Stummer et al., 2014). Along the trajectories in this study, PpIX fluorescence indicated peaks in eight measurement locations beyond, in relation to, contrast enhancement on conventional images. These volumes were classified as edema and necrosis on conventional imaging. Additionally, no PpIX-peaks were seen in four contrast-enhancing volumes along the trajectory.

### 4.3 Comparative techniques

Along the needle trajectory, no pattern between scalar values and PpIX-peaks was found, indicating that the two techniques capture different mechanisms. Accumulation of PpIX is dependent on disruption of the blood–brain barrier and altered metabolic processes in tumorous cells (af Klinteberg et al., 1999, Collaud et al., 2004, McCracken et al., 2022). Although several studies have demonstrated the correlation between PpIX fluorescence and contrast enhancement (Roberts et al., 2011, Valdés et al., 2011, Klint et al., 2023), their mechanisms of action differ. PpIX accumulates selectively in neoplastic cells, while gadolinium contrast enhancement is extracellular. Diffusion scalar maps, on the other hand, are tuned to structural tissue features such as shape, size, and orientation. Hence, all three methods have the potential to identify tumorous tissue, but rely on different underlying mechanisms, offering a plausible explanation for the lack of pattern in between PpIX-peak presence and scalar values. In dMRI, in addition to the aforementioned FA, a negative correlation between ADC and tumor cellularity has previously been observed (Nilsson et al., 2018, Chen et al., 2013, Ellingson et al., 2010). In the absence of tissue samples beyond that of the biopsy region, fluorescence and dMRI could provide constructive insights into tumor features and extent.

### 4.4 QTI vs DTI

Similar trends were observed in QTI and DTI scalars in all but two instances (AD for Patient 7 and MD for Patient 9) when processed with similar positivity constraints in the QTI+ framework. Interestingly, the values in QTI maps were consistently higher than in the DTI maps (Table S1). A contributing factor to the difference could be the entanglement of multiple contributions (shape, size, and orientation) for DTI compared to the disentangled QTI measures. This was further emphasized by the different scalar contributions in the PCA. The leading contributions of the first principal components were diffusivity (DTI) and microscopic anisotropy and kurtosis (QTI), suggesting that the two diffusion protocols capture different features. In spite of the difference in principal components, the accuracies after logic regression were similar between QTI and DTI classification of biopsy and contralateral WM data.

With respect to redundancy, the four metrics from the DTI matrix (Fig. 5D) could be reduced to two, namely FA and MD. In the QTI case, several redundancies were seen, including mutual redundancy between the three diffusivity scalars. Fig. 5C suggests similar information from diffusivity and variance, macroscopic anisotropy and orientation parameters, as well as microscopic anisotropy and kurtosis measures. Thus, several scalar subsets without redundancies can be constructed.

### 4.5 Toward clinical translation

Although the current processing demands and absence of standardized interpretation guidelines limit immediate implementation, the workflow demonstrates the feasibility of integrating quantitative diffusion metrics with optical and pathological validation in frameless navigated biopsies. In line with previous work (Szczepankiewicz et al., 2015), μFA shows promise for distinguishing tumor-associated tissues from normal-appearing WM, and its weight in the QTI principal component analysis highlights the potential of microscopic diffusion features. The redundancy analysis identified three correlated parameter groups: microscopic (μFA, C${}_{\mu }$, K${}_{\mu }$, K${}_{shear}$), macroscopic (FA, C${}_{M}$, C${}_{c}$, OP, OP2), and diffusivity (MD, AD, RD, V${}_{iso}$, V${}_{MD}$). From these, a minimal nonredundant subset could be derived to streamline future protocols, focusing for instance on μFA and MD to reduce complexity while maintaining tissue-specific contrast. Integration of these maps with conventional MRI and intraoperative fluorescence may enhance trajectory planning and sampling of heterogeneous tumor regions. The framework is compatible with standard 3 T scanners and open-source software, supporting feasibility once automated reconstruction is available. Validation in larger cohorts will be required to confirm these findings and define their role in clinical decision support.

### 4.6 Limitations

The low number of patients in this study limits the generalizability of the results, and the indications presented here need confirmation in a larger cohort. In addition, the assumption of Gaussian diffusion might not hold in tumor tissue (Alexander et al., 2019), as increased cellularity decreases the extracellular space, possibly imposing restrictions during the encoding waveform. Further, the applied diffusion sequences have a large voxel size, especially in the slice direction (2 mm DTI, 4 mm QTI), which could introduce partial volume effects. The voxels are considered particularly large compared to the 3μm tissue slabs used in the neuropathological analysis. Although the QTI maps explore the collection of environments within each voxel, the discrepancy in resolution warrants investigation in a larger cohort to elucidate the relationship between neuropathology and diffusion scalar maps. The voxel and slab resolutions should also be compared to the approximate measurement depth of the fluorescence signal (0.2–1 mm) (Lu et al., 2021) about the probe tip, as well as the biopsy sampling window of 8 mm used during surgery. The results give indications of interesting features for future non-invasive tissue analysis, but further studies are required before clinical applicability is reached.

## 5 Conclusion

Through a multimodal workflow, integrating pre-, intra-, and postoperative data, high-grade glioma biopsy tissue was associated with trends of decreased anisotropy, kurtosis, and order parameter scalar values compared to contralateral white matter, confirmed by neuropathological analysis. In contrast to DTI, the principal components of QTI demonstrate tissue separability based on microstructural features. The multimodal workflow presents a step toward clinical utility of dMRI scalars in frameless navigated brain tumor biopsies.

## CRediT authorship contribution statement

**Elisabeth Klint:** Writing – original draft, Visualization, Software, Methodology, Investigation, Formal analysis, Data curation, Conceptualization. **Johan Richter:** Writing – review & editing, Supervision, Resources, Methodology, Investigation, Conceptualization. **Teresa Nordin:** Writing – review & editing, Supervision, Methodology. **Ida Blystad:** Writing – review & editing, Methodology, Formal analysis, Data curation, Conceptualization. **Martin Hallbeck:** Writing – review & editing, Resources, Methodology, Formal analysis, Data curation, Conceptualization. **Alexandra Golby:** Writing – review & editing, Visualization, Validation, Funding acquisition, Conceptualization. **Carl-Fredrik Westin:** Writing – review & editing, Visualization, Validation, Methodology, Funding acquisition, Formal analysis, Conceptualization. **Karin Wårdell:** Writing – review & editing, Supervision, Resources, Project administration, Methodology, Investigation, Funding acquisition, Conceptualization.

## Financial support

10.13039/501100001729The Swedish Foundation for Strategic Research, Sweden (grant number RMX18-0056) provides financial support for this project. CW and AG are supported by 10.13039/100000002National Institutes of Health, United States grant R01NS125781.

## Declaration of competing interest

KW and JR have shares in the university spin-off company FluoLink AB.

## Data Availability

The data that has been used is confidential.

## Appendix A Supplementary data

MMC S1 The material includes detailed diagnoses, data for glioma feature vector calculation, and remaining QTI scalar values. MMC S1
