# Supplementary material for: Mapping brain tumor microstructure: A multimodal study of diffusion MRI, intraoperative fluorescence, and neuropathology in navigated biopsies
Source: Neuroimage Clin. 2025 Dec 12;49:103921. doi: 10.1016/j.nicl.2025.103921 (PMC12768948; doi:10.1016/j.nicl.2025.103921)
Supplement: MMC S1 — The material includes detailed diagnoses, data for glioma feature vector calculation, and remaining QTI scalar values. [file mmc1.docx]

Table S1

Patient diagnoses, neuropathological parameters, and protoporphyrin IX ﬂuorescence

| 1 | temporal | Astrocytoma | 4 | mut | meth 21 90 | | | 10 | - | - | Homozygous | + |
| --- | --- | --- | --- | --- | --- | --- | --- | --- | --- | --- | --- | --- |
|  | insular |  |  |  |  | | |  |  |  | CDKN2A/N2B del |  |
| 2 | temporal | Glioblastoma | 4 | wt | unmeth 32 60 | | | 40 | - | - - + | | |
| 3 | frontal (R) | Glioblastoma | 4 | wt | unmeth 46 40** | | | - | 60 | - - + | | |
| 4 | bifrontal | Glioblastoma | 4 | wt | unmeth 54 70 | | | - | 10 | - - + | | |
|  | transcallosal |  |  |  |  | | |  |  |  |  |  |
| 5 | frontal | Glioblastoma | 4 | wt | unmeth 4*** 20 | | | 75 | 5 | - - + | | |
|  | insular |  |  |  |  | | |  |  |  |  |  |
| 6 | frontal | Glioblastoma | 4 | wt | unmeth 50 | | 65 | 35 | - | - | - + | |
|  | basal |  |  |  |  | |  | 55 | 40 | 5 |  | |
|  | ganglia |  |  |  |  | |  | 70 | 25 | 5 |  |  |
| 7 | frontal | Glioblastoma | 4 | wt | meth | 9 | 95 | - - | | 5 | Homozygous | + |
|  | insular |  |  |  |  |  |  |  | |  | CDKN2A/N2B del |  |
| 8 fronto- Non-tumor - - - - | | | | | | | - | - - | | 100 | TERT-promotor mut  - - | |
| occipital  9 fronto Primary diﬀuse - - - 90 | | | | | | | 95 | - - | | 5 | - + | |
|  | basal | large B-cell |  |  |  |  |  |  |  |  |  |  |

Patient Location Diagnosis WHO IDH MGMT Ki67 Tumor Inﬁltrative Necrosis Non-tumor Additional PpIX grade* status status [%] [%] [%] [%] [%] markers ﬂuo

parieto-

lymphoma

*ﬂuo: ﬂuorescence; IDH: isocitrate dehydrogenase; meth: methylated; MGMT: O6-methylguanine-DNA-methyltransferase; PpIX: protoporphyrin IX; R: right; WHO: World Health Organization; wt: wildtype*

Supplementary information for *Mapping Brain Tumor Microstructure* by Klint et al

Table S2

Glioma ‘feature vectors’: mean value in biopsy volume compared to contralateral white matter (unpaired t-test corrected for type I error through false discovery rate with the Benjamini-Hochberg method)

EK, JR, TN, IB, MH, AG, CW, KW

Pat- T_1_w T2w MD AD RD FA 𝜇FA MK MKt K_𝑏𝑢𝑙𝑘_ K_𝜇_ K_𝑠ℎ𝑒𝑎𝑟_ OP OP2 V_𝑖𝑠𝑜_ V_𝑀𝐷_ V_𝑠ℎ𝑒𝑎𝑟_ C_𝑐_ C_𝑀𝐷_ C_𝑀_ C_𝜇_ PpIX Tumor Ki67 ient Gd FLAIR -peak [%] [%]

| 1 | ↑ | ↑ | 1.32 1.51 1.22 0.15 | 0.53 0.65 0.67 0.35 0.32 0.30 | 0.10 0.01 0.63 0.21 0.42 | 0.08 0.10 0.02 0.29 | + | 90 | 21 |
| --- | --- | --- | --- | --- | --- | --- | --- | --- | --- |
|  |  |  | (0.92) (1.05) (0.86) (0.13) |  |  |  |  |  |  |
|  |  |  | 0.95 1.40 0.72 0.45 | 0.78 1.15 1.36 0.41 0.95 0.74 | 0.29 0.09 0.67 0.13 0.53 | 0.35 0.12 0.22 0.61 |  |  |  |
| 2 | ↑ | ↑ | (0.61) (0.90) (0.47) (0.44)  1.41 1.68 1.28 0.19 | 0.47 0.66 0.70 0.45 0.25 0.21 | 0.14 0.02 0.66 0.33 0.33 | 0.17 0.13 0.04 0.23 | + | 60 | 32 |
| 3 | ↑ | ↑ | (0.98) (1.27) (0.90) (0.15)  1.03 1.51 0.79 0.40  0.61 0.70 0.57 0.15 | 0.71 1.05 1.20 0.48 0.73 0.58  0.54 1.06 1.09 0.71 0.38 0.36 | 0.26 0.07 0.68 0.17 0.50  0.10 0.01 0.20 0.09 0.11 | 0.31 0.14 0.17 0.51  0.10 0.19 0.03 0.30 | + | 40** | 46 |
| 4 | ↑ | ↑ | (0.49) (0.51) (0.48) (0.04)  0.98 1.28 0.84 0.34  2.15 2.32 2.06 0.10 | 0.70 1.02 1.14 0.38 0.76 0.64  0.41 0.41 0.42 0.25 0.17 0.16 | 0.21 0.05 0.59 0.15 0.44  0.07 0.01 0.99 0.36 0.63 | 0.23 0.11 0.13 0.51  0.06 0.08 0.01 0.17 | + | 70 | 54 |
|  |  |  | (1.47) (1.57) (1.42) (0.06) |  |  |  |  |  |  |
|  |  |  | 0.91 1.08 0.82 0.19 | 0.88 1.34 1.38 0.05 1.32 1.29 | 0.11 0.01 0.90 0.01 0.88 | 0.05 0.02 0.04 0.78 |  |  |  |
|  |  |  | (0.55) (0.91) (0.37) (0.51) |  |  |  |  |  |  |
| 5 | ↑ | ↑ | 1.35 1.63 1.22 0.22 | 0.49 0.87 0.92 0.60 0.32 0.27 | 0.15 0.03 0.68 0.35 0.34 | 0.22 0.16 0.06 0.26 | + | 20 | 4* |
|  |  |  | (0.94) (1.01) (0.87) (0.16) |  |  |  |  |  |  |
|  |  |  | 0.98 1.41 0.76 0.38 | 0.72 0.95 1.10 0.36 0.74 0.59 | 0.25 0.07 0.58 0.12 0.46 | 0.30 0.10 0.16 0.52 |  |  |  |
|  |  |  | (0.64) (0.96) (0.48) (0.42) |  |  |  |  |  |  |
| 6 | ↑ | ↑ | 1.16 1.41 1.03 0.22 | 0.52 0.68 0.72 0.41 0.32 0.27 | 0.16 0.03 0.48 0.19 0.29 | 0.18 0.12 0.05 0.28 | + | 65 | 50 |
|  |  |  | (0.84) (1.03) (0.74) (0.26) |  |  |  |  |  |  |
|  |  |  | 1.08 1.37 0.93 0.26 | 0.66 0.83 0.89 0.33 0.56 0.50 | 0.17 0.03 0.61 0.13 0.48 | 0.16 0.10 0.07 0.44 |  |  |  |
|  |  |  | (0.73) (0.96) (0.62) (0.29) |  |  |  |  |  |  |
| 7 | ↑ | ↑ | 0.89 1.22 0.72 0.34 | 0.74 0.83 0.93 0.20 0.74 0.63 | 0.23 0.05 0.46 0.05 0.41 | 0.22 0.06 0.12 0.54 | + | 95 | 9 |
|  |  |  | (0.66) (0.95) (0.52) (0.37) |  |  |  |  |  |  |
|  |  |  | 0.82 1.41 0.53 0.59 | 0.88 1.37 1.75 0.30 1.45 1.07 | 0.37 0.14 0.66 0.07 0.59 | 0.46 0.09 0.36 0.78 |  |  |  |
|  |  |  | (0.52) (0.89) (0.33) (0.59) |  |  |  |  |  |  |
| p |  |  | 0.16 0.47 0.08 0.03 | 0.008 0.02 0.01 0.35 0.01 0.01 | 0.04 0.04 0.47 0.08 0.07 | 0.08 0.35 0.04 0.008 |  |  |  |

(0.64) (0.99) (0.47) (0.46)

(0.61) (0.82) (0.50) (0.34)

(0.04) (0.30) (0.02) (0.002)

*Scalar maps: diﬀusivity (mean: MD, axial: AD, radial: RD), anisotropy (macroscopic: FA; microscopic:* 𝜇*FA), kurtosis (mean: MK, MKt, K*_𝑏𝑢𝑙𝑘_*, microscopic: K*_𝜇_*, K*_𝑠ℎ𝑒𝑎𝑟_*), order parameters (OP, OP2), variance (isotropic: V*_𝑖𝑠𝑜_*, V*_𝑀𝐷_*, V*_𝑠ℎ𝑒𝑎𝑟_*) and normalized scalars (C*_𝑐_ *, C*_𝑀𝐷_*, C*_𝑀_ *, C*_𝜇_*). Gd: gadolinium; FLAIR: ﬂuid attenuated inversion recovery; Ki67: proliferation index; PpIX: protoporphyrin IX*


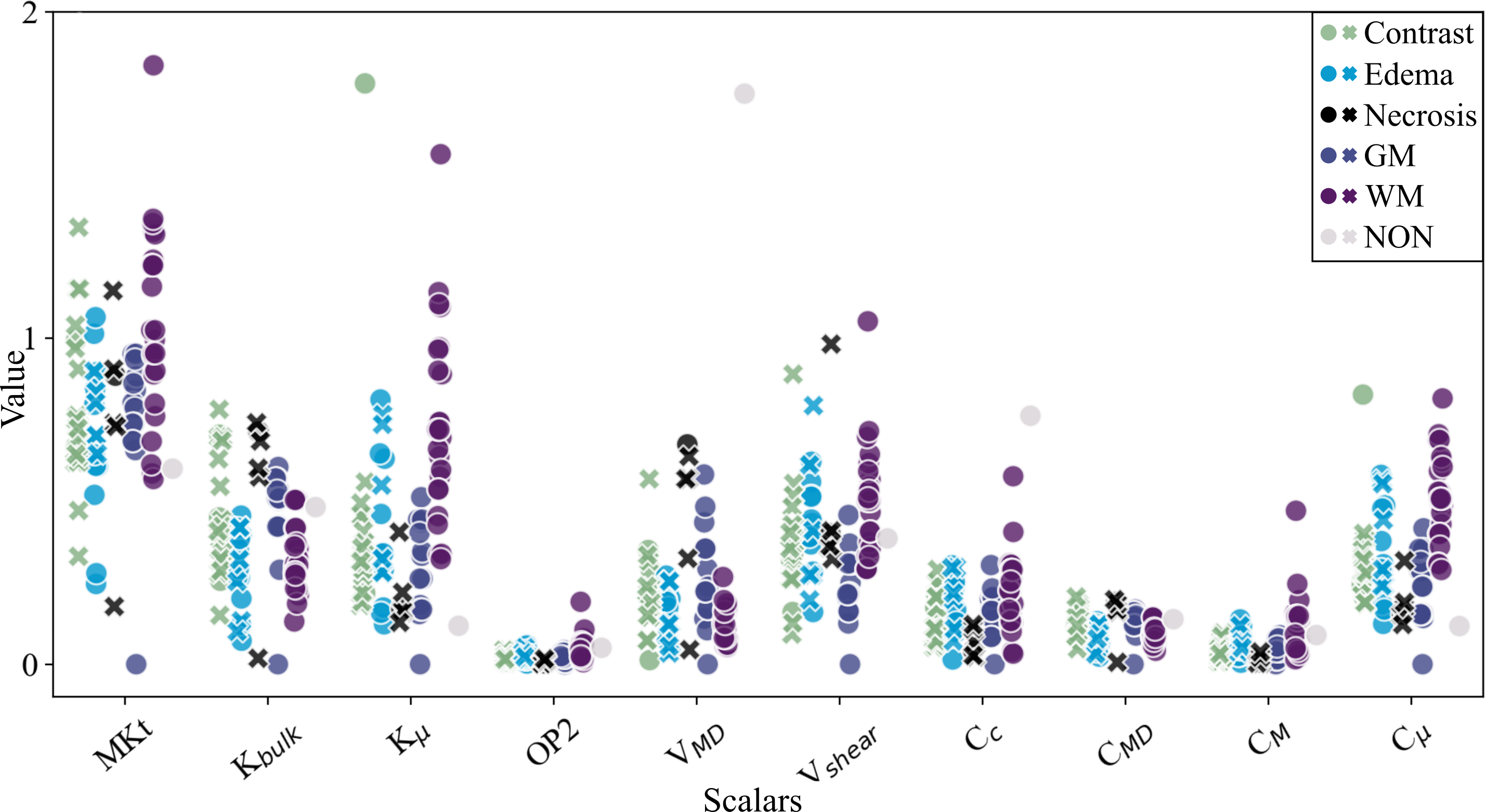


Figure S1: Mean scalar values and protoporphyrin IX ﬂuorescence in each measurement position along the trajectory for additional QTI scalars. Scalar maps: kurtosis (MKt, K_𝑏𝑢𝑙𝑘_,K_𝜇_), orientation parameters (OP2), variance (V_𝑀𝐷_, V_𝑠ℎ𝑒𝑎𝑟_), normalized scalars (C_𝑐_, C_𝑀𝐷_, C_𝑀_ , C_𝜇_). Crosses: PpIX-peaks, circles: no peak. GM: gray matter, WM: white matter.


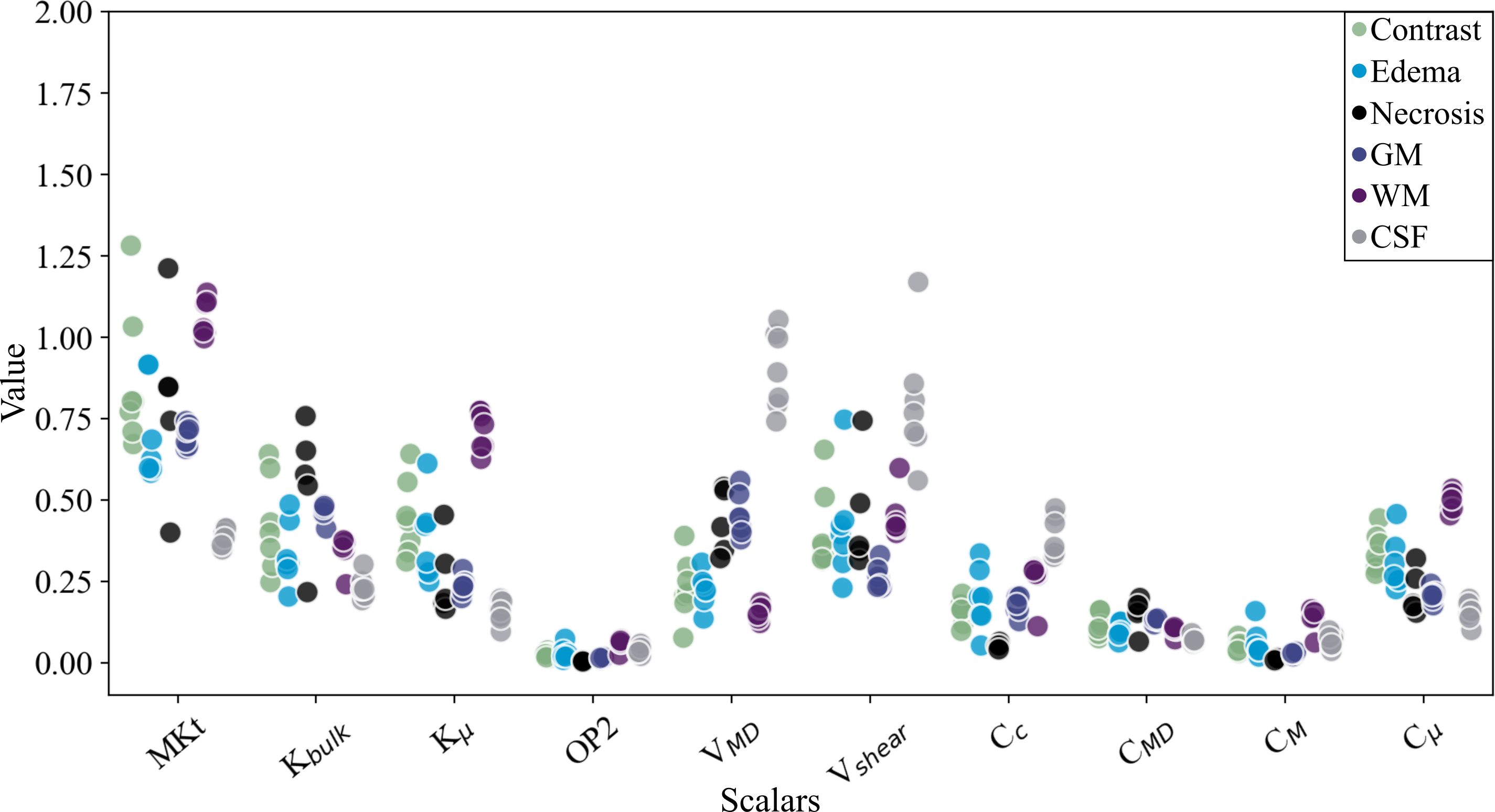


Figure S2: Mean scalar values per patient in each radiologically deﬁned tissue type (contrast-enhanced tumor, edema, necrosis, GM, WM, and CSF) for additional QTI scalars. Scalar maps: kurtosis (MKt, K_𝑏𝑢𝑙𝑘_,K_𝜇_), orientation parameters (OP2), variance (V_𝑀𝐷_, V_𝑠ℎ𝑒𝑎𝑟_), normalized scalars (C_𝑐_, C_𝑀𝐷_, C_𝑀_ , C_𝜇_). CSF: cerebrospinal ﬂuid, GM: gray matter, WM: white matter.
